# Exploration of urease-aided calcium carbonate mineralization by enzyme analyses of *Neobacillus mesonae* strain NS-6

**DOI:** 10.1128/spectrum.01891-24

**Published:** 2024-11-29

**Authors:** Zhiwei Ma, Mengyao Chen, Juncheng Lu, Shichuang Liu, Yanling Ma

**Affiliations:** 1 College of Life Science, Northwest University, Xi’an, Shaanxi, China; 2 Shaanxi Provincial Key Laboratory of Biotechnology, Key Laboratory of Resources Biology and Biotechnology in Western China, Ministry of Education, College of Life Science, Northwest University, Xi’an, Shaanxi, China; Institute of Microbiology, Chinese Academy of Sciences, Beijing, China

**Keywords:** nickel ligands, UICP, MDS, site-directed mutagenesis, western blotting

## Abstract

Urease containing nickel cofactor is crucial for urea-hydrolytic induced calcium carbonate (CaCO_3_) precipitation (UICP). However, limited information exists regarding the influence of amino acid residues interacting with nickel ions in its structure on induced CaCO_3_ mineralization. Herein, RT-qPCR was used to demonstrate that the addition of NiCl_2_ dramatically upregulated the expression of urease structural gene *ureC* that was correlated with nickel binding in *Neobacillus mesonae* strain NS-6. Homology modeling and molecular docking were employed to construct the three-dimensional structure of urease and seek the key residues involved in nickel binding process, and virtual mutation technology was adopted to inform three key residues coordinated with nickel ions and urea, His249, His275, and Asp363. Four metrics, including root mean square deviation values for mutations of those key residues in urease-urea complexes severally and wild-type, were calculated by molecular dynamics simulations when they were mutated into alanine, respectively. Subsequently, the mutations of H249A, H275A, and D363A were characterized using western blotting to reveal a decrease in the relative expression and activity of urease, along with a corresponding reduction in CaCO_3_ precipitation. Ultimately, the mutations also exhibited that they had lower substrate affinity and catalytic efficiency for urea through enzymatic properties analysis. The findings suggested that those residues played a pivotal role in UICP of strain NS-6, which would expand the theoretical basis for modulating urease activity.

**IMPORTANCE:**

Urease-producing bacterium is of great importance in diverse application fields, such as environmental remediation, due to its key driving characteristics in catalyzing urea hydrolysis via urea-hydrolytic induced CaCO_3_ precipitation (UICP). As essential cofactors of urease, nickel ions play a crucial role in regulating urease catalysis and maintaining structural stability. Numerous investigations have emphasized the impact of nickel ions on urease activity in recent years, to our best knowledge, only a few literatures have studied the molecular-level regulation of nickel-ligand residues. This study focused on the highly urease-producing bacterial *Neobacillus mesonae* NS-6 to explore the effects of specific nickel-ligand residues on the urease-aided CaCO_3_ mineralization process using molecular simulation predictions and targeted mutation experiments. The aim was to provide a molecular-level understanding of the interactive effects between urea and critical residues associated with the urease active center, as well as propose an effective modification strategy to enhance the application of UICP in future environmental areas.

## INTRODUCTION

Biomineralization is a process in which microorganisms facilitate the binding of calcium (Ca^2+^) and carbonate ions (CO_3_^2−^) to generate solid calcium carbonate (CaCO_3_) precipitates, and this biological activity is referred to as microbially induced calcite precipitation (MICP) (1, 2). As a major biologically induced mineralization process, MICP can be categorized into ureolysis (3, 4), denitrification (5), sulfate reduction (6), and other types (7, 8) based on the metabolic and chemical reactions of various microorganisms. Among them, urea-hydrolytic induced CaCO_3_ precipitation (UICP) has garnered the most widespread attention due to its high energy efficiency, low cost, and controllable reaction process (9). In the process of UICP, urease-producing bacteria catalyze the hydrolysis of urea for the formation of CaCO_3_ precipitation, which has been widely applied in various industries, including heavy metal removal (10, 11), CO_2_ sequestration (12), enhanced oil recovery (13), soil improvement (14, 15), and building restoration (16).

Urease, also known as urea amide hydrolase (EC 3.5.1.5), is a widely distributed metalloenzyme that contains nickel ions in its active site and is produced by numerous plants, fungi, bacteria, and some invertebrates (17). Bacterial urease gene clusters exhibit some differences in composition from one strain to another, with the majority comprising structural genes such as *ureA*, *ureB*, and *ureC* encoding the urease subunits, alongside accessory genes like *ureD*, *ureE*, *ureF*, and *ureG* (18). For example, urease reported in *Bacillus pasteurii* (accession no. U29368.1) (19) and *Klebsiella aerogenes* (accession no. M36068.1) (20) is composed of three subunits such as UreA(γ), UreB(β), and UreC(α), polymerized into the trimeric form [αβγ]_3_, respectively. While urease recorded in *Helicobacter pylori* (accession no. M60398.1) consists of two subunits UreA(β) and UreB(α), polymerized into the tetrameric form [(αβ)_3_]_4_ (21). On the other hand, the active site of bacterial urease necessitates the pre-insertion of nickel ions and carbamylation of lysine residues to achieve full urease activity (22). Meanwhile, the involvement of auxiliary proteins such as UreD, UreE, UreF, and UreG facilitates the transfer of nickel ions to active centers, ensuring that structural proteins are correctly folded and can efficiently catalyze urea hydrolysis (23). Previous studies have extensively elucidated the structural characteristics of bacterial ureases, as well as molecular docking has been widely applied as a prominent technique for the synthesis and development of various urease inhibitors (24, 25). Based on binding energies, virtual screening of urease inhibitors from rumen bacteria has demonstrated the stability level of relevant compounds at the urease binding site (26). Additionally, some researchers have examined the structure and function of nickel ions in urease through molecular dynamics simulations (MDS), proposing that the connection between the nickel metal center and His^α323^ was key for maintaining the flap conformation (27). Histidine residues contributing to nickel ligation were also identified by site-directed mutagenesis of urease in *K. aerogenes* (28). The aforementioned studies were of great significance for illustrating the regulation of nickel ions in urease activity, while the exploration for the role of nickel-ligand residues on the urease-aided CaCO_3_ mineralization has been rarely reported.

The genes involved in urea catabolism were elucidated through genome-wide analysis of *Neobacillus mesonae* strain NS-6 in our previous study (29). And on this basis, site-directed mutagenesis combined with homology modeling, molecular docking, virtual mutation, MDS, and enzymatic properties analysis methods was employed to predict the key residues related to nickel binding during the docking between urease and urea for investigating the potential influence of those residues on urease-aided CaCO_3_ mineralization in the current study, with aiming to provide a theoretical foundation for modulating urease activity to enhanced UICP applications in the future.

## MATERIALS AND METHODS

### Isolation and identification of *N. mesonae* strain NS-6

The urease-producing bacterial strain NS-6 was previously isolated from sandstone oil in the Ordos Basin and identified as *N. mesonae* (accession no. CP128196.1) through morphological, biochemical, and whole-genome analyses (29). The strain NS-6 was preserved in 30% glycerol at −80°C in our laboratory.

### Preparation of culture media and reagents

The Luria-Bertani (LB) broth was prepared for bacterial cultivation and pre-incubation, comprising 10.0 g/L tryptone, 5.0 g/L yeast extract, and 5.0 g/L NaCl. To assess induced CaCO_3_ precipitation, NH_4_-YE medium recommended by ATCC was used, including 10 g/L (NH_4_)_2_SO_4_, 20 g/L yeast extract, 10 g/L NaCl, 1 g/L glucose, and 20 g/L urea (30). To detect urease activity via adding nickel ions, NiCl_2_ was used as the nickel source, maintaining control amounts within the range of 0–150 μM. All culture media were adjusted to pH 7.0 and subjected to sterilization at 121°C for 20 min. Notably, prior to incorporation into the NH_4_-YE medium, urea and glucose underwent filtration through a sterile 0.45 µm membrane, while NiCl_2_ was introduced into the autoclaved NH_4_-YE medium. All organic solvents and reagents employed in this study adhered strictly to analytical grade standards.

### Urease activity and CaCO_3_ formation

Based on the Berthelot reaction, the amount of ammonia released from urea can be used to determine urease activity. One unit of urease activity is defined as the amount of enzyme required to decompose 1 µmol of urea per minute at 37°C (31). Briefly, 10 µL of appropriately diluted enzyme solution was added to 70 µL of substrate solution (30 g/L urea in 50 mM citric acid-sodium citrate buffer, pH 6.0). The mixture was incubated at 37°C for 20 min and subsequently terminated by adding 40 µL of 10% trichloroacetic acid. Following this, 40 µL each of chromogenic reagent I (0.125 g sodium nitroprusside and 3 g phenol dissolved in 50 mL ultrapure water) and chromogenic reagent II (1.5 mL NaClO and 2.625 g NaOH dissolved in 50 mL ultrapure water) were added to the reaction mixture and incubated for an additional 20 min (32). Finally, the absorbance was measured at 625 nm. Each experiment was performed in triplicate.

To investigate and quantify CaCO_3_ formation induced by strain NS-6, 3.0 mL of stabilized-phase bacterial cells (1% vol/vol) were introduced into 300 mL of NH_4_-YE medium supplemented with 2% urea and 25 mM CaCl_2_, as well as different concentrations of NiCl_2_ ranging from 0 to 150 µM. The mixture was incubated at 30°C and 200 rpm for 48 h severally, and then a 30-mL aliquot of every culture was extracted. After that, the sample was centrifuged at 2,650 × *g* for 3 min to separate the supernatant, and the resultant precipitated CaCO_3_ was collected, respectively (29). Any residual cells and culture components were eliminated by filtering the precipitate, followed by rinsing with deionized water and anhydrous ethanol. Subsequently, every precipitate was subjected to vacuum drying at 80°C for 24 h and then weighed. For further analysis of CaCO_3_, it was washed with HCl solution, dried, and weighed once more. The quantity of CaCO_3_ was determined by calculating the difference in mass between the two drying steps.

### RT-qPCR of urease structural gene expression

Following the manufacturer’s instructions, 1 mL of TRIzol was utilized for isolating and extracting total RNA from strain NS-6. Then RNA concentration was determined at 260 nm using a Nanodrop spectrophotometer, and RNA quality was assessed based on the OD_260/280_ absorbance ratio (within the range of 1.8–2.0). Total RNA was reverse transcribed into cDNA employing the NovoeScript RT kit with synthetic primers listed in Table S1, wherein *rpsl* was chosen as the internal reference gene. To ensure primer specificity, validation was conducted through PCR reactions, resulting in the observation of bands of the correct size on agarose gels, as depicted in Fig. S1. Furthermore, the results were quantitatively analyzed using the 2^−△△CT^ method with fluorescence quantitative PCR (Quant Gene 9600, Hangzhou) (33).

### Template-based homology modeling and molecular docking of urease

The urease protein sequence for strain NS-6 was obtained from the whole-genome data. The automated SWISS-MODEL server was utilized to predict and generate the three-dimensional (3D) structure of urease based on Global Model Quality Estimate (GMQE) and Qualitative Model Energy Analysis (QMEAN) values (34). PyMOL software was applied to visualize the model and compare conformational disparities with the template. The homologous modeling model was evaluated in PDB format using the SAVES server (https://saves.mbi.ucla.edu/), incorporating ERRAT, PROCHECK, and WATCHCHECK.

Meanwhile, molecular docking as a computer method was employed to seek the interactions between urease and urea in strain NS-6. The 3D structure of urea was downloaded from the PubChem database (https://pubchem.ncbi.nlm.nih.gov/) in SDF format and converted to PDB format using OpenBabel. The urease and ligand urea underwent pre-processing utilizing PyMOL and AutoDock Tools-1.5.6. The cubic grid box used for blind docking had dimensions of 126 Å (*x*, *y*, *z*) with a spacing of 0.731 Å. The docking process was conducted employing default parameters, and the binding free energy was determined using the Lamarckian genetic algorithm. The final conformation was selected based on the complex structure with the lowest binding free energy (35). Moreover, molecular docking results were analyzed using PyMOL and LIGPLOT. Following the principle of alanine scanning, virtual mutations were then performed on non-conserved sites within the urea range by Discovery Studio software to predict the residues related to nickel binding.

### MDS of mutant urease-urea docking complexes

The optimal molecular docking results were employed for MDS analysis of the urease-urea complexes. Gromacs 2020.3 was utilized, employing the aber99sb-ildn.ff molecular force field, and the TIP3P water model was used to parameterize urease and urea within a cubic water box. Then, complex systems were constructed, and these systems underwent energy minimization, followed by 100 ps of equilibrium kinetics under NVT and NPT ensembles, respectively. The V-rescale method was selected to control the system temperature, while the Berendsen method was employed to maintain pressure. Due to the presence of nickel ions, restrictive MDS were performed using 100 ns sampling at a simulated temperature (300K) and pressure (1 bar), with the integration step set to 2 fs. After the simulation, the root mean square deviation (RMSD), root mean square fluctuation (RMSF), radius of gyration (RG), and hydrogen bonds were calculated using built-in modules within the Gromacs program.

### Site-directed mutagenesis of targeted amino acids in *ureC* gene

Based on molecular docking and virtual mutation analysis, the predicted key residues in the UreC domain of urease were further confirmed by site-directed mutation into Ala, respectively. The *ureC* target fragment containing the mutation site was obtained through a three-step PCR reactionwith the primers listed in Table S1. Briefly, mutation primers H249A-F/H249A-R and template amplification primers *ureC*-F/*ureC*-R were employed to produce the *ureC* target fragments including the mutation site *ureC*-F/H249A-R and H249A-F/*ureC*-R by PCR 1. The high-fidelity enzymes were used for PCR 2 on the equal mixture of the above recovered purified fragments, followed by PCR 3 with *ureC*-F/*ureC*-R primers using this system as a template. Subsequently, the *ureC* target fragment containing the mutation site was subjected to double digestion with the plasmid vector pET-28a (kanamycin-resistant) using the restriction endonucleases *Sal* I and *Nhe* I, and ligation with T4 DNA ligase to generate recombinant plasmids pET-28a-H249A. The mutation of His275 and Asp363 to Ala was achieved by a similar strategy (36). Following the manufacturer’s instructions, the DNA purification and recovery kit (Tiangen, Beijing) was utilized to recover the pertinent fragments during the mutation process. The accuracy of the results was assessed through agarose gel electrophoresis and sequence alignment conducted using SnapGene.

### Western blotting of mutant UreC protein

The mutants successfully identified through sequencing were chemically transformed into *Escherichia coli* BL21 (DE3) and incubated overnight at 37°C with agitation at 180 rpm in LB medium supplemented with kanamycin. The cultures were grown under identical conditions until reaching an OD_600_ value of 0.6–0.8. Subsequently, urease protein expression was induced by adding IPTG at a final concentration of 0.5 mg/mL. The parameters for ultrasonic cell disruption were set as follows: 20% power, 3 s ultrasonic treatment with a 2.7-s interval, totaling 30 min. The membrane was incubated overnight at 4°C with anti-urease antibody (1:1,000) after being blocked with 5% Blotting Grade solution for 2 h. Followed by three washes with TBST for 10 min each, the membrane was incubated with HRP-goat anti-rabbit IgG antibody (1:2,000) at room temperature for 2 h, in which β-actin was chosen as the internal reference. Protein expression was quantitatively analyzed using a chemiluminescence imaging system, with subsequent analysis of the results performed utilizing ImageJ.

### Purification and biochemical characterization of mutant protein

The mutants were purified using a Ni-NTA affinity column, and the purification results were identified by polyacrylamide-gel electrophoresis (SDS-PAGE). Following the manufacturer’s instructions, the protein concentration was measured employing the Bradford protein assay kit (Elabscience, China). To determine the kinetic parameters of the mutants, urease activity was measured using various substrate concentrations (5–80 mM urea) under standard assay conditions. The standard Michaelis−Menten equation was fitted to the data using Origin 2021. To investigate the optimal reaction temperature of the mutants, purified urease samples were properly diluted and incubated at temperatures ranging from 30°C to 80°C for 30 min to assess their relative activity. Additionally, relative activity was measured by incubating the purified urease in citrate-sodium citrate buffer (50 mM, pH 4.0–6.0), Tris-HCl buffer (50 mM, pH 7.0–8.0), and glycine-sodium hydroxide buffer (50 mM, pH 9.0–11.0) at 4°C for 6 h, followed by measurement under standard conditions to determine the optimal reaction pH. Each experiment was performed in triplicate.

## RESULTS AND DISCUSSION

### Appropriate NiCl_2_ increased the expression of urease structural genes

**Fig 1:**
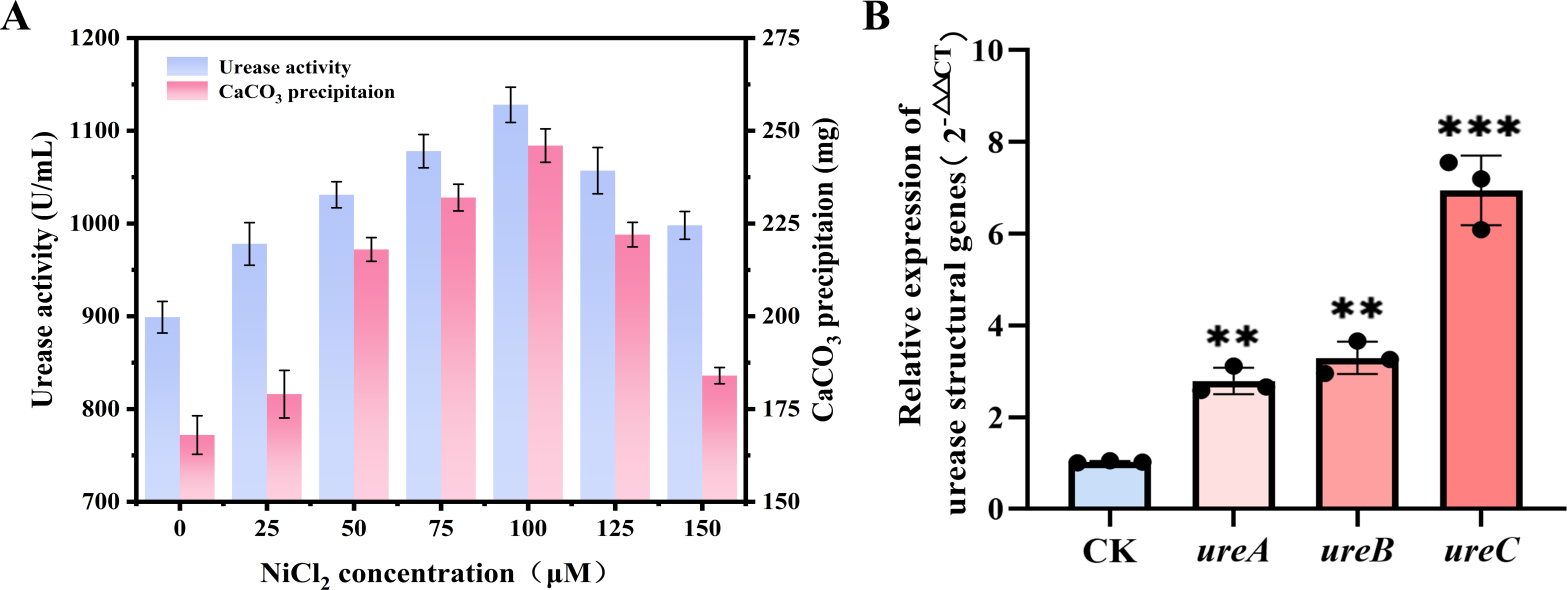
Effect of nickel ions on urease activity and CaCO_3_ precipitation at different NiCl_2_ concentrations (**A**) and the expression of urease structural genes (B, thereinto “* *” indicated *P* < 0.01, “* * *” showed *P* < 0.001) in strain NS-6.

Various concentrations of NiCl_2_ ranging from 0 to 150 µM were introduced into the NH_4_-YE medium to investigate their impact on strain NS-6 biomineralization, and the culture broth was sampled to assess urease activity and CaCO_3_ precipitation after 32 h (29). The results displayed that NiCl_2_ concentrations below the threshold of 100 µM augmented urease activity and CaCO_3_ precipitation to some extent compared to the control group, but excessive concentrations of nickel ions above 100 µM could potentially inhibit both urease activity and biomineralization in strain NS-6 (Fig. 1A). These results prompted the selection of 100 µM NiCl_2_ for further RT-qPCR analysis, aiming to determine how nickel impacted the expression of crucial urease genes in strain NS-6. According to our previously genome-wide annotation of strain NS-6, the related information of urease structural genes *ureA*, *ureB*, and *ureC* was retrieved for primer synthesis. Compared to the control group without adding NiCl_2_, the expression levels of those genes were upregulated (Fig. 1B). Notably, *ureC* exhibited the most significant upregulation, potentially attributed to the presence of the nickel ion active site within its α subunit. Thus, UreC was hypothesized to represent the primary binding site for nickel ions in regulating the combination of nickel ions with the active center of urease (37).

As a cofactor of urease, the presence of nickel ions significantly facilitated the maturation and activity of urease. This process stabilized negative charges through promoting ligand binding with certain functional groups in the substrate, which is essential for maintaining the correct folding and conformation of urease, thereby facilitating the catalytic reaction (38, 39). Although existing literature researches have investigated how nickel influenced the transcription of urease genes in *H. pylori*, particularly noting the concurrent upregulation of *ureA* and *ureB* (40, 41), there remained a notable gap in research regarding the regulatory effects of nickel on the expression levels of urease structural proteins. Herein, the expression of the urease structural gene *ureC* in strain NS-6 was significantly upregulated with the addition of NiCl_2_, consistent with the previous expression analysis of urease genes in *Proteus mirabilis* (42). Unlike earlier studies that predominantly concentrated on urease activity as influenced by nickel, the results herein provided a comprehensive evaluation of how nickel ions specifically regulate the expression of urease structural genes. Moreover, this systematic verification of the unique impact of nickel on urease gene expression indicated nickel not only as an enhancer of urease activity, but also as a pivotal regulatory factor. Such insights highlighted the significance of *ureC* in promoting urease activity and suggested avenues for following research into the molecular exploration by which nickel ions exert their regulatory effects.

### The specific nickel-ligand residues (His249, His275, and Asp363) were screened during the docking process

**Fig 2:**
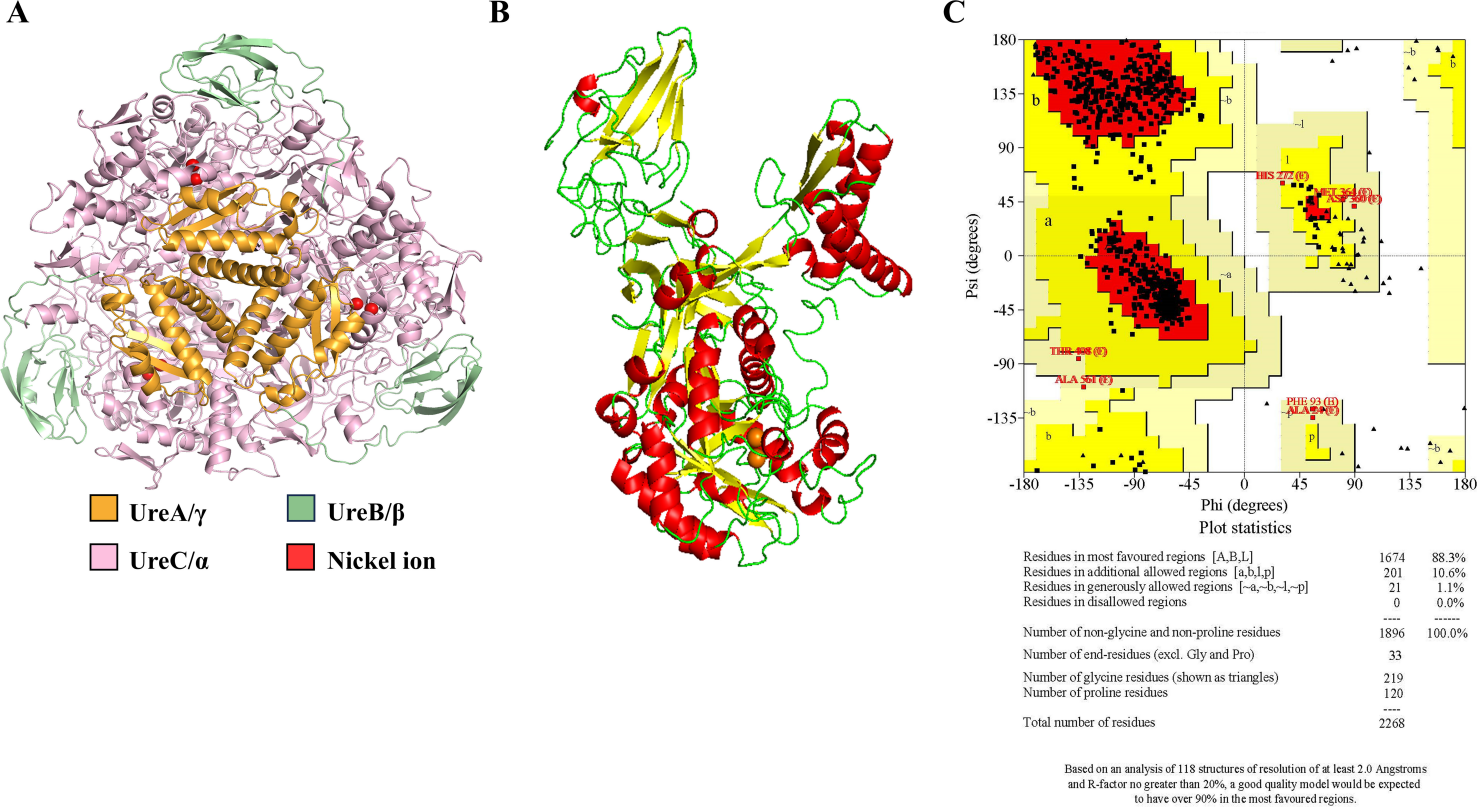
Homology modeling of urease in strain NS-6, including 3D modeling model (**A**), secondary structure of urease monomer (B, thereinto: red, yellow, and green denoted *α*-helices, *β*-sheets, and random coils, respectively), as well as Ramachandran plot (C, thereinto: red, positive yellow, light yellow, and white areas showed allowable, extra allowable, loose, and non-allowable areas, respectively).

To observe the interaction between urease and urea more intuitively, the model (PDB ID: 1fwe.1) that exhibited the best GMQE and QMEAN values among the options available on the SWISS-MODEL server was chosen as an appropriate template for homology modeling. It could be seen that the urease structure of strain NS-6 presented by visualization was a typical trimer form [αβγ]_3_ (Fig. 2A), and the secondary structure of an individual monomer was also depicted in Fig. 2B. Subsequently, a 3D model of urease from *K. aerogenes* was retrieved from the Protein Data Bank (PDB), wherein the spatial positional disparities and superimposition between this model and the constructed 3D structure of urease in strain NS-6 were evaluated using PYMOL software (Fig. S2). Although the structures of the two proteins did not exactly align, the observed angle difference indicated that the proteins were in a nearly parallel orientation in space. To assess the rationality of the constructed 3D structure, the Ramachandran analysis conducted by the PROCHECK server revealed the number of residues in the allowable, additional allowable, loose, and non-allowable regions was 1,674, 201, 21, and 0, correspondingly (Fig. 2C). Additionally, the overall quality factor obtained from the ERRAT server was 95.3% (Fig. S3A), and the superior quality and high reliability of the model were further demonstrated in the WATCHCHECK server (Fig. S3B).

**Fig 3:**
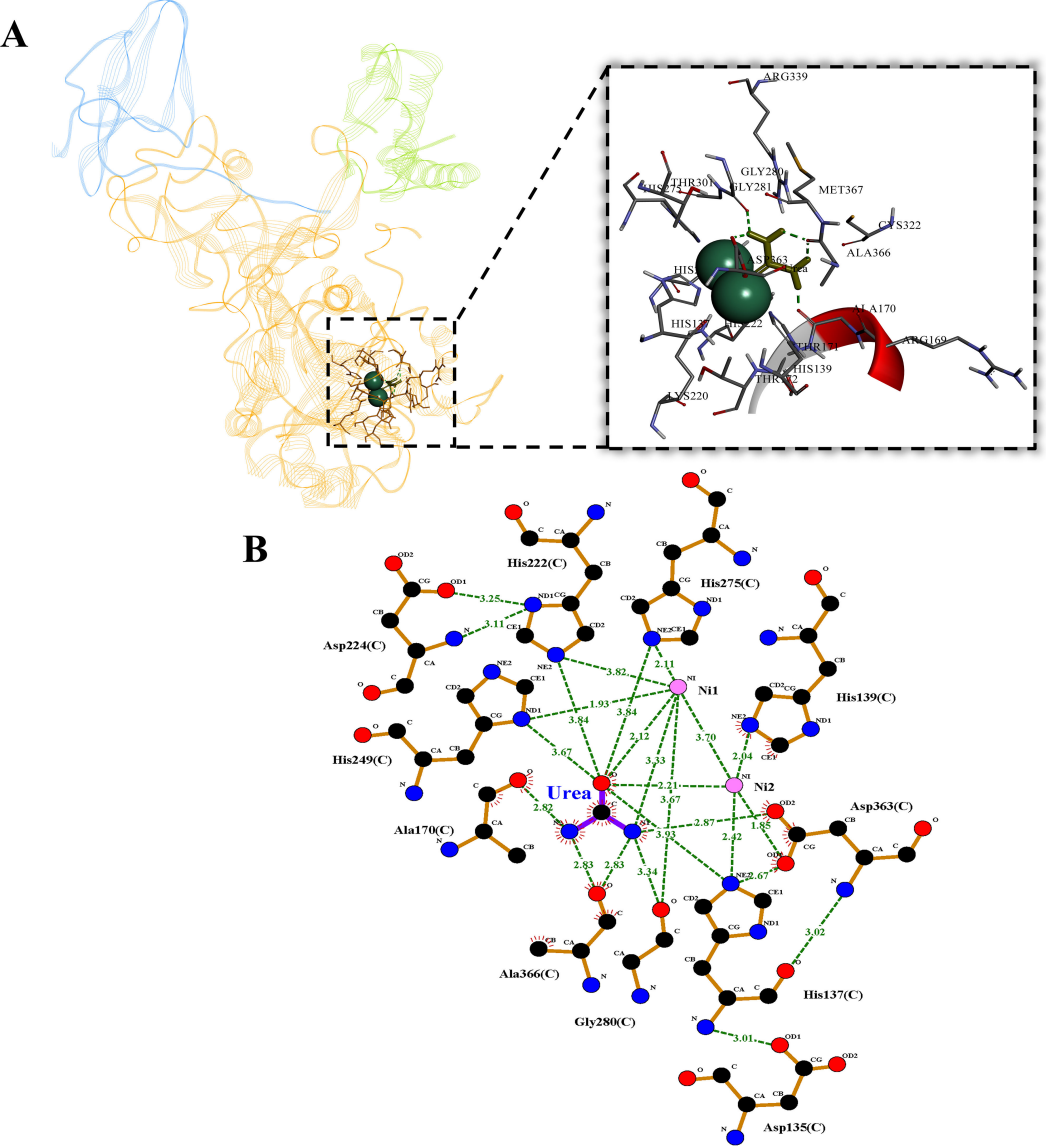
The binding state of urea and nickel ion active site, composed of molecular docking 3D schematic diagram (**A**) and 2D plane interaction diagram (B, thereinto green line represented hydrogen bond interaction force).

To further investigate nickel-ligand residues, molecular docking with urea using a preconstructed 3D model of urease was conducted in strain NS-6, in which the ligand was set as semi-flexible while maintaining the receptor rigid, as well as docking iterations were performed 50 times and the method yielding the lowest binding energy was selected. The docking results revealed that dual nickel ion active sites were situated within the conserved subunit UreC and bound to urea (Fig. 3A). Based on the “bridging hydroxide mechanism” hypothesis, the presence of a dual nickel center in urease facilitated the formation of hydroxide ion, an essential auxiliary substrate for the urea hydrolysis reaction, thereby enhancing the binding efficiency and catalytic activity toward urea (37). LIGPLOT analysis for visualizing information more clearly demonstrated that urea interacted with dinuclear nickel through hydrogen bonding, with distance of 3.7 nm between the dual nickel ions (Fig. 3B). Previous studies have reported that there might be two potential binding modes of monodentate and bidentate during the docking process of nickel ions with urea. While the nickel ions in urease of strain NS-6 were inclined to bind to urea in a more stable bidentate mode, potentially explaining the high efficiency and stability of strain NS-6 urease in inducing CaCO_3_ mineralization (43, 44). Meanwhile, except for His139 being coordinated solely to the nickel ion along with Ala170 and Ala366 being coordinated exclusively to urea, the remaining six amino acids were all exhibited to be coordinated with both nickel ions and urea, including His137, His222, His249, His275, Gly280, and Asp363. These residues might play crucial roles in the binding and stabilization of enzyme-substrate complex interactions and urease activity. Moreover, virtual mutation analysis revealed elevated mutation energies for the remaining six residues excluding Gly280, meaning a declined urease binding capacity to urea after mutation (Fig. S4). Therefore, His249, His275, and Asp363 with the highest mutation energy and coordination with nickel ions and urea were selected to explore the potential influence of those residues on the urease-aided CaCO_3_ mineralization.

### Predicted nickel-ligand residues showed vital role in urease activity

**Fig 4:**
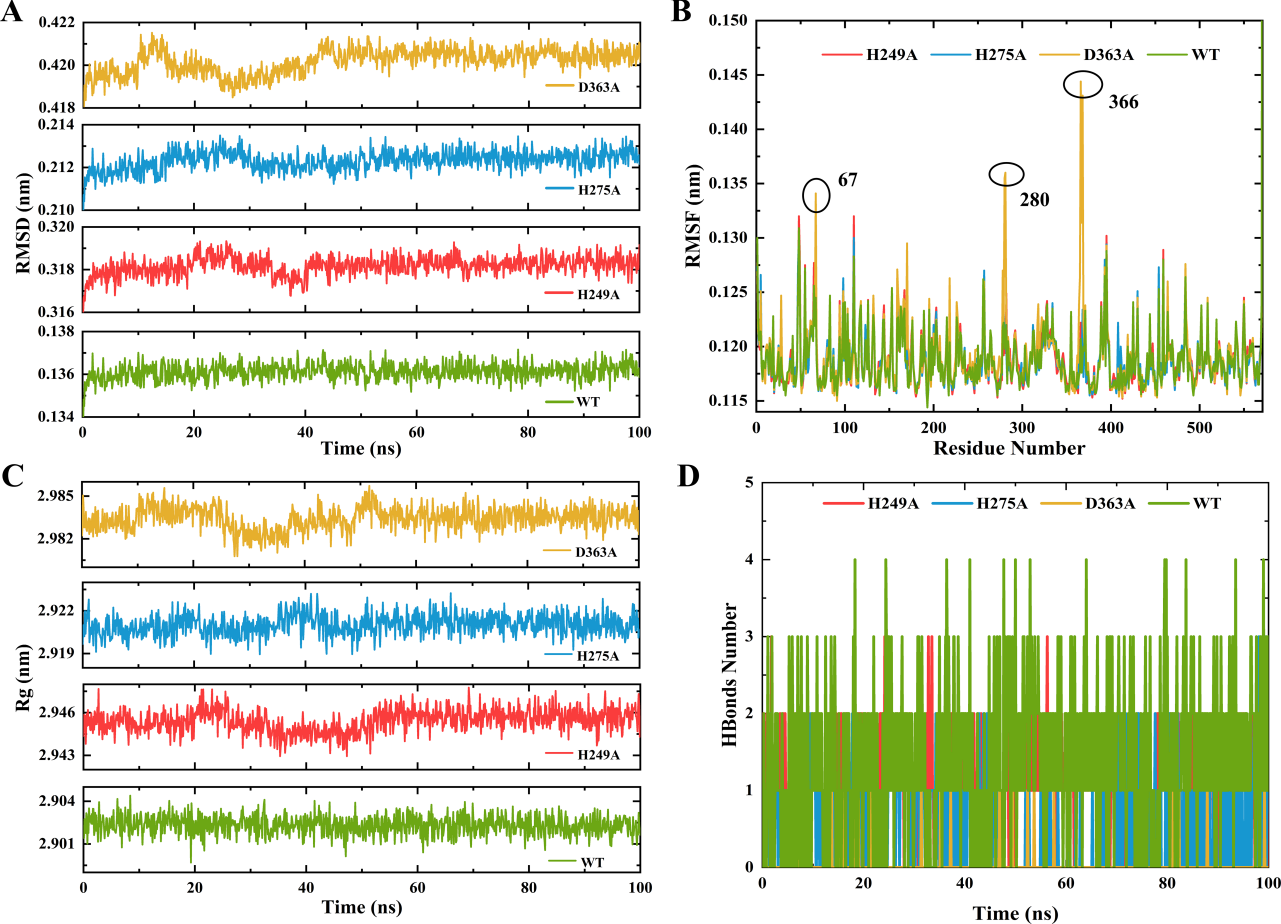
MDS analysis of urease-urea complexes in D363A (yellow), H275A (blue), H249A (red), and WT (green), containing individual analyses of RMSD (**A**), RMSF (**B**), Rg (**C**), and hydrogen bond (**D**).

After mutating His249, His275, and Asp363 to Ala using PyMOL, four parameters (RMSD, RMSF, Rg, and hydrogen bonds) were calculated by MDS to analyze the stability of urease-urea complexes including WT, H249A, H275A, and D363A during 100 ns (45, 46). Typically, higher RMSD values indicate more pronounced structural alterations in the protein system (47). The three mutant complexes exhibited fluctuations in the range of 0–50 ns, and the mean RMSD values for WT, H249A, H275A, and D363A were 0.136, 0.318, 0.212, and 0.420 nm, respectively (Fig. 4A), implying that those mutant proteins had elevated instability compared to that of the WT during the simulation process. The ascending RMSF values for the H249A, H275A, and D363A mutants compared to that of WT, with exceptions noted at certain residues such as sites 67, 280, and 366, suggesting that those mutations contributed to the partial unfolding of urease (Fig. 4B). In addition, the Rg values of four proteins ranging from 2.901 to 2.984 nm, while the protein from WT demonstrated a denser structure compared to the other three mutant proteins (Fig. 4C). In the meantime, Fig. 4D illustrated the continuous detection of hydrogen bonding changes in the complexes throughout the 100 ns simulation, wherein the number of hydrogen bonds fluctuated between 1 and 4 over time, indicating that the protein from WT formed more hydrogen bonds than those proteins from H249A, H275A, and D363A severally.

**Fig 5:**
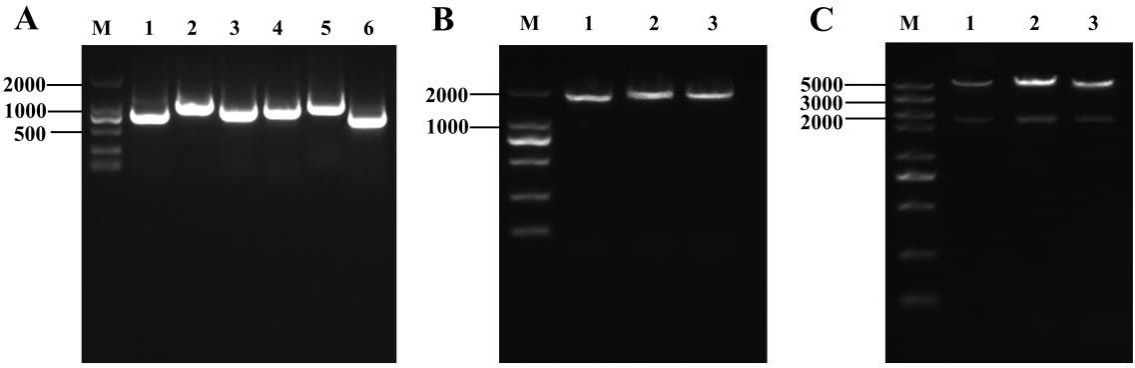
Targeted mutation vector construction, comprising successive amplification of the upstream and downstream fragments of the mutant (A, thereinto M meant marker, odd number expressed as upstream fragments of the mutant H249A, H275A, and D363A, even number indicated as downstream fragments of the mutant H249A, H275A, and D363A), the complete fragment of the mutant (B, thereinto 1–3 represented *ureC* fragment containing the mutant H249A, H275A, and D363A, respectively), as well as double enzyme digestion test of recombinant plasmid (C, thereinto 1–3 showed orderly double-enzyme digestion products of pET-28a-H249A, pET-28a-H275A, and pET-28a-D363A).

**Fig 6:**
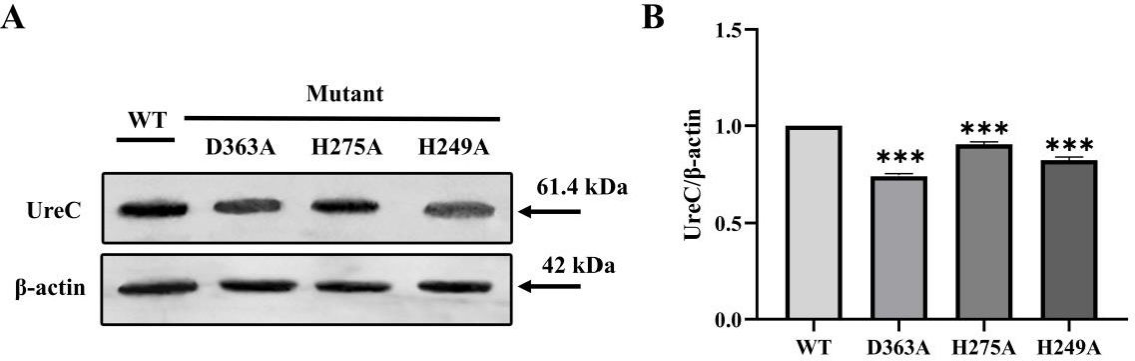
Western blotting validation of key residues for banding of WT and mutant D363A, H275A, and H249A of UreC (**A**), and statistical analysis of UreC expression levels in WT and mutant D363A, H275A, and H249A, respectively (B, thereinto “* * *” indicated *P* < 0.001).

To further verify the pivotal role of the three residues (His249, His275, and Asp363), overlap extension PCR-mediated site-directed mutagenesis was employed to introduce a single mutant base into the *ureC* gene fragment. After three PCR amplifications, the agarose gel electrophoresis results were displayed that the specific bands in both upstream, downstream, and *ureC* destination fragments of the mutation sites corresponding to His249, His275, and Asp363 with the anticipated size (Fig. 5A and B). And Fig. 5C illustrated the verification process of the recombinant plasmid pET-28a-H249A, pET-28a-H275A, and pET-28a-D363A within the *E. coli* DH5α culture through double enzyme digestion, respectively, in which an observed specific band of approximately 1,713 bp was indicated the successful digestion process. Meanwhile, the results demonstrated that all sequences remained unchanged, except for His249, His275, and Asp363 that were effectively substituted to Ala, meaning the successful mutation (Fig. S5). Moreover, the expression levels of the mutant proteins H249A, H275A, and D363A were determined by western blotting analysis to confirm the mutation outcomes. As illustrated in Fig. 6A, the immunoreactive bands of the anticipated size (approximately 61.4 kDa) were observed in both the control group (WT) and the experimental group (mutant types), thus validating the successful expression of the mutant proteins. Specifically, the protein accumulation level of the mutant D363A was significantly reduced, implying a notable effect of the Asp363 mutation on UreC protein expression (Fig. 6B). In comparison to urease activity of WT (1,522 ± 34 U/mg), reductions of 39.6%, 23.9%, and 54.5% in urease activity were noted for H249A, H275A, and D363A induced and purified under standard culture conditions (Fig. S6), concomitant with a corresponding decrease in CaCO_3_ precipitation, respectively (Table 1). These findings highlighted the significance of the amino acid binding domain associated with the nickel ion active site on the urease-aided CaCO_3_ mineralization.

**TABLE 1:** Urease activity and CaCO_3_ precipitation in WT and mutant types^*a*^

Type	Urease activity (U/mg)	CaCO_3_ precipitation (mg)
WT	1,522 ± 34	162 ± 5
H249A	920 ± 28	112 ± 6
H275A	1,158 ± 37	139 ± 4
D363A	692 ± 23	89 ± 5

^*a*^ The results shown are the mean ± standard deviation of three independent experiments.

Currently, the utilization of mutation analysis based on computer simulations facilitates the precise prediction of the impact of site-specific mutations on protein structure, stability, and activity. This was consistent with previous research involving mutation of individual amino acids during the docking process of *Glycine max* and *Medicago truncatula* urease with urea identified Arg435 and Arg437 as potential hotspot residues capable of modifying enzyme activity or substrate specificity (48). On the other hand, the stability of the urease-urea complex can be effectively analyzed using MDS, which was once elucidated the pivotal roles of Ser324, Ala329, and Val385 within the rice urease binding to the substrate urea in the study of rice urease function (34). Similarly, the combination condition between three mutation residues in strain NS-6 urease and urea was analyzed via MDS in our investigation, confirming the detrimental effects induced of mutations on the urease-urea docking complexes. With regard to targeted mutagenesis experiments about substrate-bound amino acids and nickel-linked, cysteines were previously identified at the center of urease activity in *K. aerogenes* (49), but few subsequent reports have been published. In this instance, laboratory-based site-directed mutagenesis was adopted to verify that these specific nickel-ligand residues could influence urease activity and CaCO_3_ precipitation during the urease-aided CaCO_3_ mineralization, meaning a pivotal role for these residues in the binding process of urease to urea and potentially influencing substrate binding and catalytic activity.

### The combination of urease mutants with urea exhibited differences in enzymatic properties

To further investigate the effect of residues coordinated with nickel ions on substrate binding and catalytic activity, WT and mutant ureases (H249A, H275A, and D363A) purified by nickel column were utilized to analyze their optimal reaction temperature and pH. As depicted in Fig. S7A, urease activity increased faster with rising temperatures within the range of 30–40°C. Above 50°C, the three mutants exhibited lower urease activity compared to the WT. At 70°C, the urease activity of the WT exceeded that of H249A, H275A, and D363A by factors of 1.37, 1.33, and 1.5, respectively, underscoring its superior performance under elevated temperature conditions. Furthermore, the optimal reaction pH of both WT and mutant ureases was approximately 8.0 (Fig. S7B). Within the pH range of 5–10, the WT demonstrated superior urease activity compared to the mutants, but D363A showed higher urease activity at pH 4 and 11. These findings suggested that mutations in specific residues could alter the active site, substrate binding region, or reaction properties of strain NS-6 urease, thereby influencing its enzyme activity and functional characteristics under elevated temperature conditions, while also enhancing its structural integrity and stability under extreme pH conditions. In addition to determining the optimal reaction temperature and pH, kinetic parameters (*K_m_*, *V*_*max*_, *K*_*cat*_, and *K*_*cat*_/*K_m_*) were also calculated by fitting nonlinear curve to the dataunder standard assay conditions (Fig. S7C; Table 2). All three mutants exhibited higher *K_m_* values and lower *K*_*cat*_*/K_m_* values compared to the WT. In other words, mutations involving nickel-ligand residues reduced the affinity and catalytic efficiency of strain NS-6 urease for urea.

**TABLE 2:** Kinetic parameters of strain NS-6 urease and its mutants

Type	*K_m_* (mM)	*V*_*max*_ (μmol mg^−1^ min^−1^)	*K*_*cat*_ (s^−1^)	*K*_*cat*_ */K_m_* (s^−1^ M^−1^)
WT	8.53 ± 1.20	2,047.49 ± 57.40	3,270.75 ± 91.69	(3.8 ± 0.1) × 10^5^
H249A	9.96 ± 0.92	787.19 ± 17.87	1,813.80 ± 41.18	(1.8 ± 0.1) × 10^5^
H275A	8.97 ± 0.95	1,070.84 ± 22.74	2,543.56 ± 54.01	(2.9 ± 0.1) × 10^5^
D363A	12.02 ± 2.13	744.39 ± 34.53	1,528.52 ± 70.90	(1.3 ± 0.1) × 10^5^

The analysis of enzymatic properties, such as enzyme kinetics, offers valuable insights into the pivotal roles enzymes play in environmental applications. In recent years, there has been an increasing number of studies investigating the enzymatic properties of mutant proteins (50, 51). However, knowledge regarding the enzymatic information of urease mutants remains relatively scarce. Kinetic parameters and enzyme stability of *Bacillus paralicheniformis* urease (BpUrease) mutants have been previously determined, confirming that site-saturation mutagenesis (SSM) can enhance the ability of BpUrease to degrade ethyl carbamate (31). Similarly, the enzymatic properties of strain NS-6 urease mutants H249A, H275A, and D363A were analyzed in this study, indicating that the residues coordinated with nickel ions likely play a key role in the UICP process. Furthermore, based on the previous research on *H. pylori* urease mutants (52), it is evident that single-site mutation has limited impact on the pocket configuration of enzyme activity site. Future studies should consider cumulative mutations to further expand evolutionary potential and enhance its efficacy in urease. This approach aims to widen the applicability of the urease-aided CaCO_3_ mineralization for environmental fields.

### Conclusions

In summary, this study elucidated the structural formation of specific nickel-ligand residues His249, His275, and Asp363 in the UreC domain of urease in *N. mesonae* strain NS-6, as well as the stability of the corresponding docking complexes through molecular docking and MDS. Results from western blotting and enzymatic properties analyses of mutants following laboratory-based site-directed mutagenesis further revealed that these residues were crucial for urease binding to urea. The combined utilization of computational simulations and experimental methods provided a more comprehensive understanding of the effects of key residues related to active sites on the urease-aided CaCO_3_ mineralization, addressing the challenge of how UICP can enhance urease activity and improve CaCO_3_ formation efficiency in environmental applications, thereby offering valuable insights for exploring UICP at the molecular level.

## Data Availability

The raw whole-genome reads of strain NS-6 has been deposited in GenBank with accession no. CP128196.1.

## SUPPLEMENTAL MATERIAL

Supplemental table and figures spectrum.01891-24-s0001.pdf Table S1; Fig. S1 to S7.

ASM does not own the copyrights to Supplemental Material that may be linked to, or accessed through, an article. The authors have granted ASM a non-exclusive, world-wide license to publish the Supplemental Material files. Please contact the corresponding author directly for reuse.

## References

[1] Chen J, Liu B, Zhong M, Jing C, Guo B. 2022. Research status and development of microbial induced calcium carbonate mineralization technology. PLOS ONE 17:e0271761. doi:10.1371/journal.pone.0271761 35867666 PMC9334024

[2] Seifan M, Berenjian A. 2019. Microbially induced calcium carbonate precipitation: a widespread phenomenon in the biological world. Appl Microbiol Biotechnol 103:4693–4708. doi:10.1007/s00253-019-09861-5 31076835

[3] Naveed M, Duan J, Uddin S, Suleman M, Hui Y, Li H. 2020. Application of microbially induced calcium carbonate precipitation with urea hydrolysis to improve the mechanical properties of soil. Ecol Eng 153:105885. doi:10.1016/j.ecoleng.2020.105885

[4] Tobler DJ, Cuthbert MO, Greswell RB, Riley MS, Renshaw JC, Handley-Sidhu S, Phoenix VR. 2011. Comparison of rates of ureolysis between Sporosarcina pasteurii and an indigenous groundwater community under conditions required to precipitate large volumes of calcite. Geochim Cosmochim Acta 75:3290–3301. doi:10.1016/j.gca.2011.03.023

[5] Wang Z, Su J, Ali A, Gao Z, Zhang R, Li Y, Yang W. 2023. Microbially induced calcium precipitation driven by denitrification: Performance, metabolites, and molecular mechanisms. J Environ Manage 338:117826. doi:10.1016/j.jenvman.2023.117826 37001427

[6] Li C, Wang Y, Zhou T, Bai S, Gao Y, Yao D, Li L. 2019. Sulfate acid corrosion mechanism of biogeomaterial based on MICP technology. J Mater Civ Eng 31:04019097. doi:10.1061/(ASCE)MT.1943-5533.0002695

[7] Abdel-Basset R, Hassan EA, Grossart H-P. 2020. Manifestations and environmental implications of microbially-induced calcium carbonate precipitation (MICP) by the cyanobacterium Dolichospermum flosaquae. Biogeosciences Discuss. doi:10.5194/bg-2020-378

[8] Zúñiga-Barra H, Ostojic C, Torres-Aravena Á, Rivas M, Vílchez C, Jeison D. 2024. Use of photosynthetic MICP to induce calcium carbonate precipitation: prospecting the role of the microorganism in the formation of CaCO3 crystals. Algal Res 80:103499. doi:10.1016/j.algal.2024.103499

[9] Anbu P, Kang C-H, Shin Y-J, So J-S. 2016. Formations of calcium carbonate minerals by bacteria and its multiple applications. Springerplus 5:250. doi:10.1186/s40064-016-1869-2 27026942 PMC4771655

[10] Chen S, Hu X, Lv Y, Zhu X, Liu X, Zhang M. 2022. Isolation of urease-producing bacteria and removal effect on the Cd and Pb in filtrate. Adv Eng Technol Res 1:225. doi:10.56028/aetr.1.1.225

[11] Jiang N-J, Liu R, Du Y-J, Bi Y-Z. 2019. Microbial induced carbonate precipitation for immobilizing Pb contaminants: toxic effects on bacterial activity and immobilization efficiency. Sci Total Environ 672:722–731. doi:10.1016/j.scitotenv.2019.03.294 30974362

[12] Clarà Saracho A, Marek EJ. 2024. Uncovering the dynamics of urease and carbonic anhydrase genes in ureolysis, carbon dioxide hydration, and calcium carbonate precipitation. Environ Sci Technol 58:1199–1210. doi:10.1021/acs.est.3c06617 38173390

[13] Song C, Elsworth D. 2020. Microbially induced calcium carbonate plugging for enhanced oil recovery. Geo Fluids 2020:1–10. doi:10.1155/2020/5921789

[14] Lai H-J, Cui M-J, Chu J. 2023. Effect of pH on soil improvement using one-phase-low-pH MICP or EICP biocementation method. Acta Geotech 18:3259–3272. doi:10.1007/s11440-022-01759-3

[15] Tang C-S, Yin L, Jiang N, Zhu C, Zeng H, Li H, Shi B. 2020. Factors affecting the performance of microbial-induced carbonate precipitation (MICP) treated soil: a review. Environ Earth Sci 79:94. doi:10.1007/s12665-020-8840-9

[16] Hao L, Lin P, Garg A. 2022. Unsaturated soil properties of MICP treated granitic residual soil of Shantou region of China. Acta Geophys 71:1885–1894. doi:10.1007/s11600-022-00967-5

[17] Yang W, Peng Z, Wang G. 2023. An overview: metal-based inhibitors of urease. J Enzyme Inhib Med Chem 38:361–375. doi:10.1080/14756366.2022.2150182 36446640 PMC11003495

[18] Kappaun K, Piovesan AR, Carlini CR, Ligabue-Braun R. 2018. Ureases: historical aspects, catalytic, and non-catalytic properties - a review. J Adv Res 13:3–17. doi:10.1016/j.jare.2018.05.010 30094078 PMC6077230

[19] You J-H, Song B-H, Kim J-G, Lee M-H, Kim S-D. 1995. Genetic organization and nucleotide sequencing of the ure gene cluster in Bacillus pasteurii. Mol Cells 5:359–369. doi:10.1016/S1016-8478(23)17336-5

[20] Mulrooney SB, Hausinger RP. 1990. Sequence of the Klebsiella aerogenes urease genes and evidence for accessory proteins facilitating nickel incorporation. J Bacteriol 172:5837–5843. doi:10.1128/jb.172.10.5837-5843.1990 2211515 PMC526901

[21] Labigne A, Cussac V, Courcoux P. 1991. Shuttle cloning and nucleotide sequences of Helicobacter pylori genes responsible for urease activity. J Bacteriol 173:1920–1931. doi:10.1128/jb.173.6.1920-1931.1991 2001995 PMC207722

[22] Mazzei L, Cianci M, Benini S, Bertini L, Musiani F, Ciurli S. 2016. Kinetic and structural studies reveal a unique binding mode of sulfite to the nickel center in urease. J Inorg Biochem 154:42–49. doi:10.1016/j.jinorgbio.2015.11.003 26580226

[23] Nim YS, Wong K-B. 2019. The maturation pathway of nickel urease. Inorganics 7:85. doi:10.3390/inorganics7070085

[24] Kafarski P, Talma M. 2018. Recent advances in design of new urease inhibitors: a review. J Adv Res 13:101–112. doi:10.1016/j.jare.2018.01.007 30094085 PMC6077125

[25] Loharch S, Berlicki Ł. 2022. Rational development of bacterial ureases inhibitors. Chem Rec 22:e202200026. doi:10.1002/tcr.202200026 35502852

[26] Zhao S, Togtokhbayar N, Narantuya B. 2022. Virtual screening for the discovery of novel urease inhibitors of rumen bacterial urease. Biochemistry. doi: 10.1101/2022.08.16.504210

[27] Lv J, Jiang Y, Yu Q, Lu S. 2011. Structural and functional role of nickel ions in urease by molecular dynamics simulation. J Biol Inorg Chem 16:125–135. doi:10.1007/s00775-010-0711-5 20890717

[28] Park IS, Hausinger RP. 1993. Site-directed mutagenesis of Klebsiella aerogenes urease: identification of histidine residues that appear to function in nickel ligation, substrate binding, and catalysis. Protein Sci 2:1034–1041. doi:10.1002/pro.5560020616 8318888 PMC2142404

[29] Xu R, Zhang S, Ma Z, Rao Q, Ma Y. 2023. Characterization and genome analysis of Neobacillus mesonae NS-6, a ureolysis-driven strain inducing calcium carbonate precipitation. Front Microbiol 14:1277709. doi:10.3389/fmicb.2023.1277709 38029179 PMC10646308

[30] Zhao Y, Xiao Z, Lv J, Shen W, Xu R. 2019. A novel approach to enhance the urease activity of Sporosarcina pasteurii and its application on microbial-induced calcium carbonate precipitation for sand. Geomicrobiol J 36:819–825. doi:10.1080/01490451.2019.1631911

[31] Liu Q, Yao X, Liang Q, Li J, Fang F, Du G, Kang Z. 2018. Molecular engineering of Bacillus paralicheniformis acid urease to degrade urea and ethyl carbamate in model chinese rice wine. J Agric Food Chem 66:13011–13019. doi:10.1021/acs.jafc.8b04566 30450906

[32] Liu Q, Chen Y, Yuan M, Du G, Chen J, Kang Z. 2017. A Bacillus paralicheniformis iron-containing urease reduces urea concentrations in rice wine. Appl Environ Microbiol 83:e01258-17. doi:10.1128/AEM.01258-17 28646111 PMC5561274

[33] Lian D, Xu Y, Deng Q, Lin X, Huang B, Xian S, Huang P. 2019. Effect of patchouli alcohol on macrophage mediated Helicobacter pylori digestion based on intracellular urease inhibition. Phytomedicine 65:153097. doi:10.1016/j.phymed.2019.153097 31568921

[34] Kumar A, Kumar S, Kumar A, Sharma N, Sharma M, Singh KP, Rathore M, Gajula MNVP. 2018. modeling, molecular docking and molecular dynamics based functional insights into rice urease bound to ureahttps://doi.org/10.1007/s40011-017-0898-0. Proc Natl Acad Sci India 88:1539–1548. doi:10.1007/s40011-017-0898-0

[35] He Y, Zhang X, Li M, Zheng N, Zhao S, Wang J. 2022. Coptisine: a natural plant inhibitor of ruminal bacterial urease screened by molecular docking. Sci Total Environ 808:151946. doi:10.1016/j.scitotenv.2021.151946 34843773

[36] Xue S-W, Tian Y-X, Pan J-C, Liu Y-N, Ma Y-L. 2021. Binding interaction of a ring-hydroxylating dioxygenase with fluoranthene in Pseudomonas aeruginosa DN1. Sci Rep 11:21317. doi:10.1038/s41598-021-00783-9 34716364 PMC8556375

[37] Mazzei L, Musiani F, Ciurli S. 2020. The structure-based reaction mechanism of urease, a nickel dependent enzyme: tale of a long debate. J Biol Inorg Chem 25:829–845. doi:10.1007/s00775-020-01808-w 32809087 PMC7433671

[38] Hu HQ, Huang H-T, Maroney MJ. 2018. The Helicobacter pylori HypA·UreE2 complex contains a novel high-affinity Ni(II)-binding site. Biochemistry 57:2932–2942. doi:10.1021/acs.biochem.8b00127 29708738 PMC6260798

[39] Tsang KL, Wong K-B. 2022. Moving nickel along the hydrogenase-urease maturation pathway. Metallomics 14:mfac003. doi:10.1093/mtomcs/mfac003 35556134

[40] Benoit SL, Maier RJ. 2011. Mua (HP0868) is a nickel-binding protein that modulates urease activity in Helicobacter pylori. MBio 2:e00039-11. doi:10.1128/mBio.00039-11 21505055 PMC3086059

[41] Nolden L, Beckers G, Möckel B, Pfefferle W, Nampoothiri KM, Krämera R, Burkovskia A. 2000. Corynebacterium glutamicum: organization of corresponding genes and investigation of activity. FEMS Microbiol Lett 189:305–310. doi:10.1111/j.1574-6968.2000.tb09248.x 10930756

[42] Ramadevi S, Kaleeswaran B, Ilavenil S, Upgade A, Tamilvendan D, Rajakrishnan R, Alfarhan AH, Kim Y-O, Kim H-J. 2020. Effect of traditionally used herb Pedalium murex L. and its active compound pedalitin on urease expression – For the management of kidney stone. Saudi J Biol Sci 27:833–839. doi:10.1016/j.sjbs.2020.01.014 32127759 PMC7042614

[43] Saito T, Takano Y. 2022. QM/MM molecular dynamics simulations revealed catalytic mechanism of urease. J Phys Chem B 126:2087–2097. doi:10.1021/acs.jpcb.1c10200 35238572 PMC8935366

[44] Jabri E, Carr MB, Hausinger RP, Karplus PA. 1995. The crystal structure of urease from Klebsiella aerogenes. Science 268:998–1004. doi:10.1126/science.7754395 7754395

[45] Chan L-C, Mat Yassim AS, Ahmad Fuaad AAH, Leow TC, Sabri S, Radin Yahaya RS, Abu Bakar AMS. 2023. Inhibition of SARS-CoV-2 3CL protease by the anti-viral chimeric protein RetroMAD1. Sci Rep 13:20178. doi:10.1038/s41598-023-47511-z 37978223 PMC10656507

[46] Fatriansyah JF, Rizqillah RK, Yandi MY, Fadilah, Sahlan M. 2022. Molecular docking and dynamics studies on propolis sulabiroin-A as a potential inhibitor of SARS-CoV-2. J King Saud Univ Sci 34:101707. doi:10.1016/j.jksus.2021.101707 34803333 PMC8591974

[47] Mir WR, Bhat BA, Rather MA, Muzamil S, Almilaibary A, Alkhanani M, Mir MA. 2022. Molecular docking analysis and evaluation of the antimicrobial properties of the constituents of Geranium wallichianum D. Don ex Sweet from Kashmir Himalaya. Sci Rep 12:12547. doi:10.1038/s41598-022-16102-9 35869098 PMC9307801

[48] Filiz E, Vatansever R, Ozyigit II. 2016. Molecular docking of Glycine max and Medicago truncatula ureases with urea; bioinformatics approaches. Mol Biol Rep 43:129–140. doi:10.1007/s11033-016-3945-7 26852122

[49] Martin PR, Hausinger RP. 1992. Site-directed mutagenesis of the active site cysteine in Klebsiella aerogenes urease. J Biol Chem 267:20024–20027. doi:10.1016/S0021-9258(19)88659-3 1400317

[50] Abdel-Naby MA, El-Wafa WMA, Salem GEM. 2020. Molecular characterization, catalytic, kinetic and thermodynamic properties of protease produced by a mutant of Bacillus cereus-S6-3. Int J Biol Macromol 160:695–702. doi:10.1016/j.ijbiomac.2020.05.241 32485254

[51] Almahasheer AA, Mahmoud A, El-Komy H, Alqosaibi AI, Aktar S, AbdulAzeez S, Borgio JF. 2022. Novel feather degrading keratinases from Bacillus cereus group: biochemical, genetic and bioinformatics analysis. Microorganisms 10:93. doi:10.3390/microorganisms10010093 35056542 PMC8781890

[52] Hendricks JK, Mobley HL. 1997. Helicobacter pylori ABC transporter: effect of allelic exchange mutagenesis on urease activity. J Bacteriol 179:5892–5902. doi:10.1128/jb.179.18.5892-5902.1997 9294450 PMC179482

