# Supplementary material for: Exploration of urease-aided calcium carbonate mineralization by enzyme analyses of Neobacillus mesonae strain NS-6
Source: Microbiol Spectr. 2024 Nov 29;13(1):e01891-24. doi: 10.1128/spectrum.01891-24 (PMC11705952; doi:10.1128/spectrum.01891-24)
Supplement: Supplemental table and figures — Table S1; Fig. S1 to S7. [file spectrum.01891-24-s0001.pdf]

Supplementary Information

Exploration of urease-aided calcium carbonate mineralization by enzyme analyses of *Neobacillus mesonae* strain NS-6

Zhiwei Ma [a, b], Mengyao chen [a, b], Juncheng Lu [a, b], Shichuang Liu [a, b], Yanling Ma [a, b]*

[a] College of Life Science, Northwest University, 229 Tai bai North Rd, Xi'an, Shaanxi 710069, China

[b] Shaanxi Provincial Key Laboratory of Biotechnology, Key Laboratory of Resources Biology and Biotechnology in Western China, Ministry of Education, College of Life Science, Northwest University, Xi'an, Shaanxi 710069, China

* Corresponding Author: Yanling Ma

Email: mayanling@nwu.edu.cn, TEL: 86-29-13572806395, FAX: 86-29-88303572,

Present/permanent Address: 229 Tai bai North Rd, Xi'an, Shaanxi 710069, China

Legends to the supplementary figures

Figure S1. Validation of primers for RT-qPCR analysis (Lane M: Marker; Lane 1: *ureA*; Lane 2: *ureB*; Lane 3: *ureC*; Lane 4: *rpsl* as positive control). The gel image showed a single band with an expected size of about 100 bp, indicating successful amplification of the target DNA fragment.

Figure S2. Three-dimensional structure of urease monomer (A) and template (B) as well as conformation comparison (C).

Figure S3. Homology model evaluation using ERRAT (A) and WHATCHECK of urease in strain NS-6 (B), respectively.

Figure S4. Virtual mutation of non-conserved amino acids around urea.

Figure S5. Comparison of sequencing before and after site-directed mutagenesis of the *ureC* gene. Red positions indicate successful mutation of His249 (A), His275 (B), and Asp363 (C) to alanine, respectively.

Figure S6. SDS-PAGE analysis of the purified urease-UreC of WT (A) and mutant H249A (B), H275A (C), and D363A (D). (Lane M: Marker; Lane 1: supernatant after crushing; Lane 2: flow through; Lane 3: wash; Lane 4: purified protein).

Figure S7. Optimal reaction temperature (A), pH (B) and Michaelis-Menten curves (C) analysis of WT and mutant H249A, H275A, and D363A.

Table S1 Primers used in this study[a]

Gene	Forward primer (5'-3')	Reverse primer (5'-3')
q-*ureA*	CGGCAGATCTTGCAAAGAGG	GGTGATAAGGGCTACTGCCT
q-*ureB*	AACCGGGCGATGAGAAAGAG	TTGAGCCGTCCGTTTTGTTG
q-*ureC*	GGCGGATACCGACTTGTTCA	CGCCAAAAACGACTTCCTCG
q-*rpsl*	GCGGTGGCTACACTGGTCAA	CGTATTTCTTACGCTCTTTCATACG
ureC	GACGTCGACTGCATGAGTTTTGAAATGTCAAG	CGCGCTAGCGCGTCAAAATAGAAAATAACGCT
H249A	CGACGTACAGGTGGCACTT<u>GCA</u>GCCGACACGTTAAATGAAG	CTTCATTTAACGTGTCGGC<u>TGC</u>AAGTGCCACCTGTACGTCG
H275A	GGGTTATTCACATGTAC<u>GCA</u>ACAGAAGGTGCGGGCGG	CCGCCCGCACCTTCTGT<u>TGC</u>GTACATGTGAATAACCC
D363A	GTATGACAAGTTCTG<u>CG</u>GCTCAGGCAATGGGC	GCCCATTGCCTGAGC<u>CG</u>CAGAACTTGTCATAC

[a]The underline indicates the mutation site.

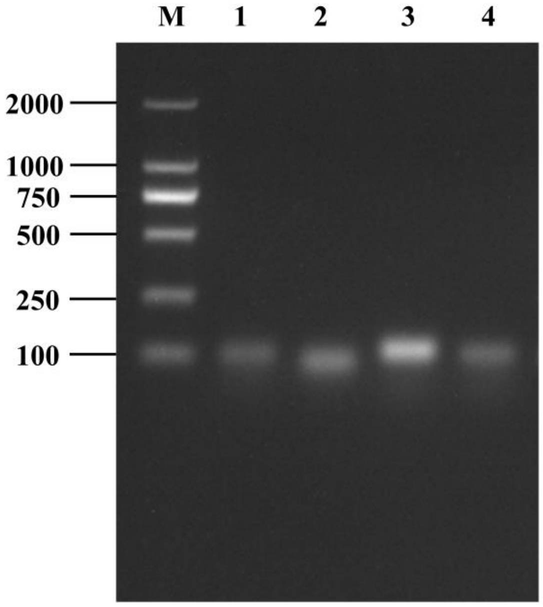

Figure S1. Validation of primers for RT-qPCR analysis (Lane M: Marker; Lane 1: *ureA*; Lane 2: *ureB*; Lane 3: *ureC*; Lane 4: *rpsl* as positive control). The gel image showed a single band with an expected size of about 100 bp, indicating successful amplification of the target DNA fragment.

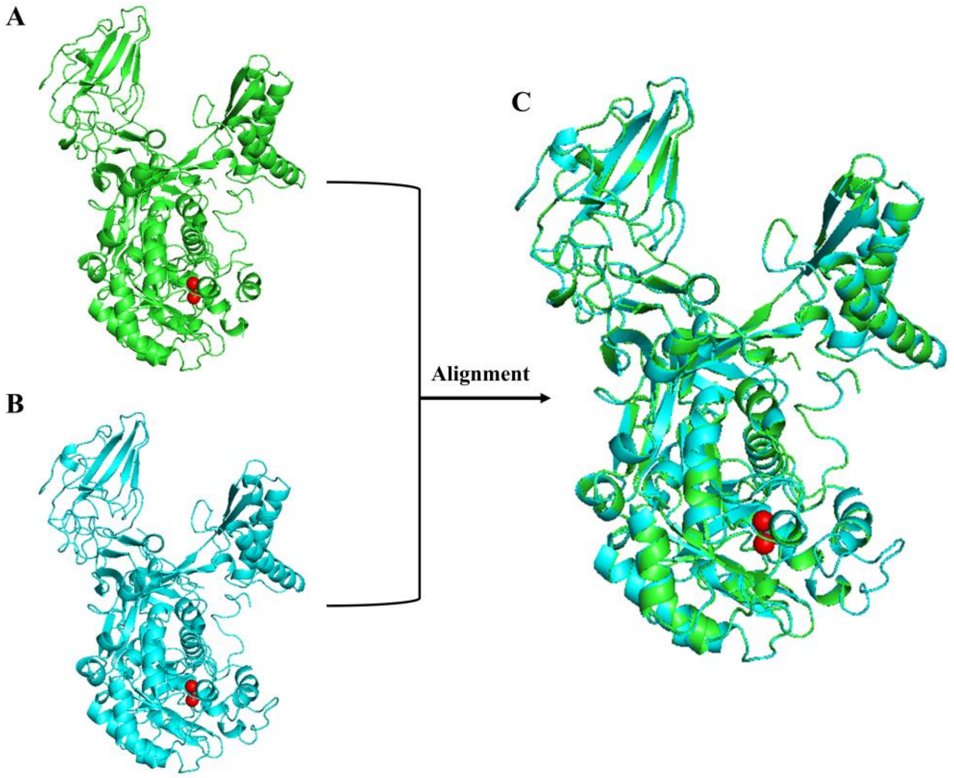

Figure S2. Three-dimensional structure of urease monomer (A) and template (B) as well as conformation comparison (C).

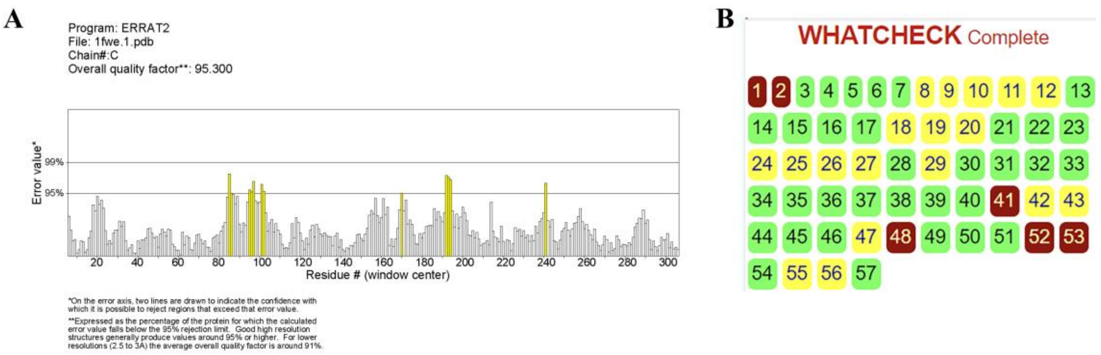

Figure S3. Homology model evaluation using ERRAT (A) and WHATCHECK of urease in strain NS-6 (B), respectively.

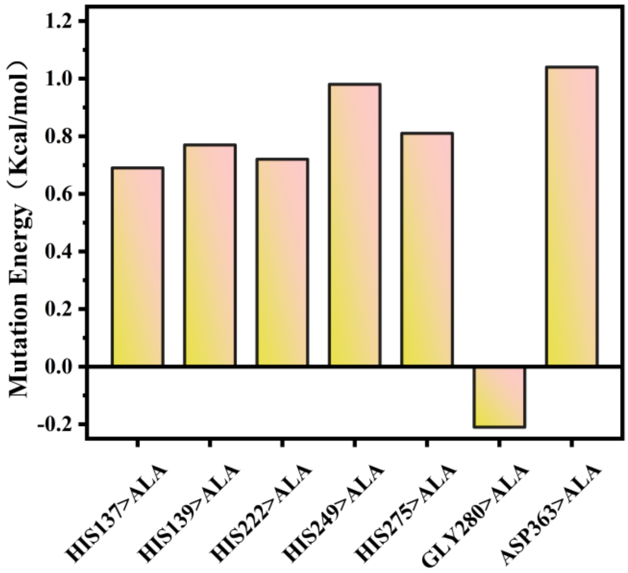

Figure S4. Virtual mutation of non-conserved amino acids around urea.

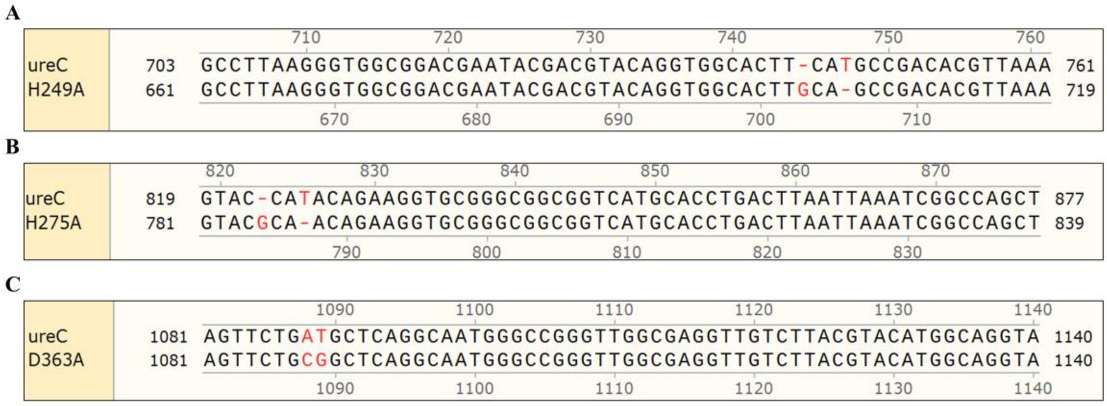

Figure S5. Comparison of sequencing before and after site-directed mutagenesis of the *ureC* gene. Red positions indicate successful mutation of His249 (A), His275 (B), and Asp363 (C) to alanine, respectively.

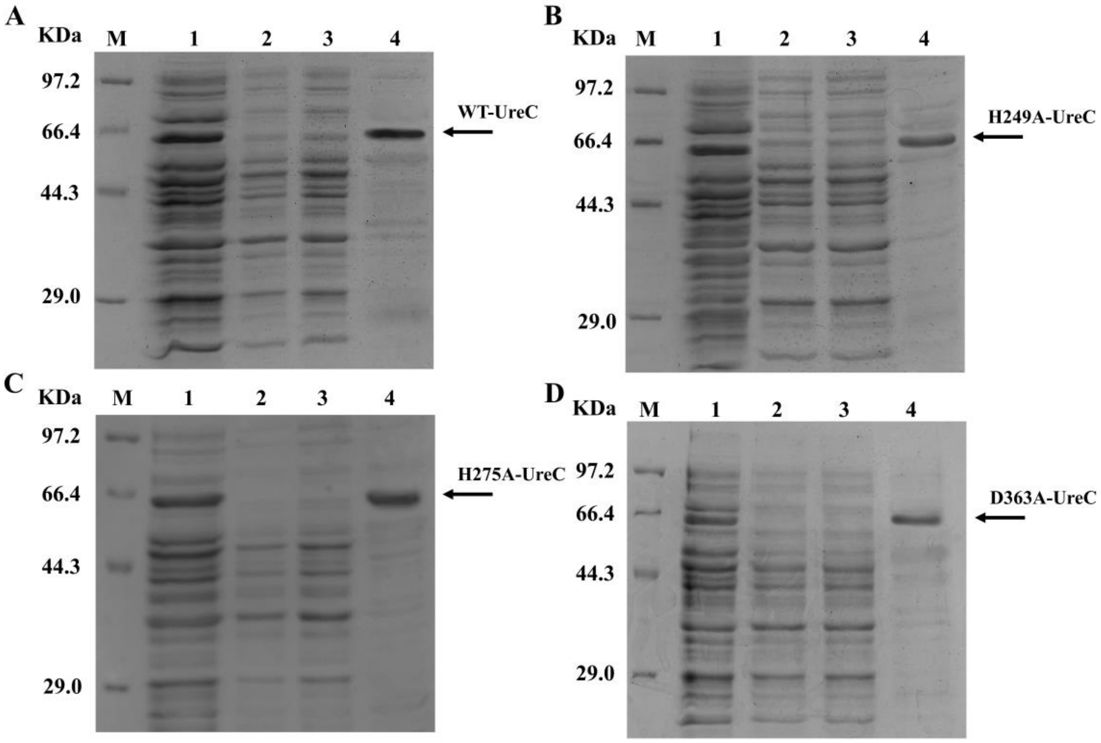

Figure S6. SDS-PAGE analysis of the purified urease-UreC of WT (A) and mutant H249A (B), H275A (C), and D363A (D). (Lane M: Marker; Lane 1: supernatant after crushing; Lane 2: flow through; Lane 3: wash; Lane 4: purified protein).

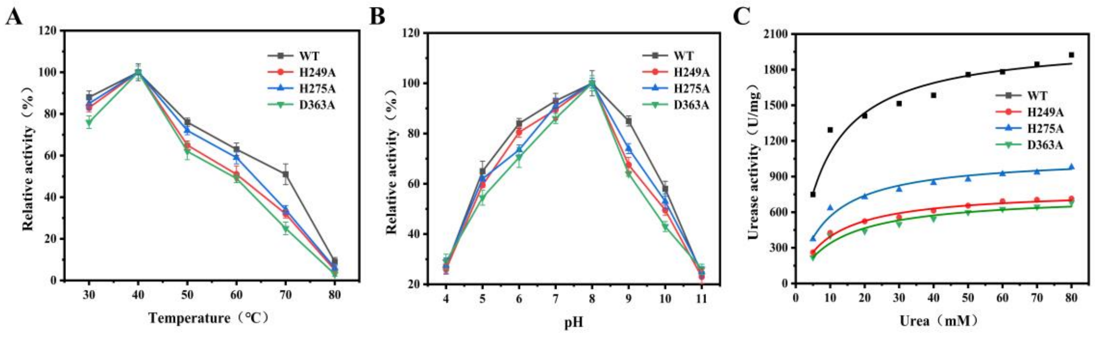

Figure S7. Optimal reaction temperature (A), pH (B) and Michaelis-Menten curves (C) analysis of WT and mutant H249A, H275A, and D363A.
